# The Optimal Timing of Platelet-Rich Plasma (PRP) Injection for Nerve Lesion Recovery: A Preliminary Study

**DOI:** 10.1155/2022/9601547

**Published:** 2022-05-06

**Authors:** Muhammad Pandunugrahadi, Komang Agung Irianto, Oen Sindrawati

**Affiliations:** ^1^Orthopaedic and Traumatology Department, Dr Soetomo General Hospital/Faculty of Medicine, Airlangga University, Surabaya, Indonesia; ^2^Pathologic Anatomy Department, Faculty of Medicine, Widya Mandala Catholic University, Surabaya, Indonesia

## Abstract

**Introduction:**

Without appropriate treatment, nerve injuries may result in permanent loss of function. Platelet-rich plasma (PRP) injection is found to help in nerve regeneration. PRP is a concentrated platelet derived from autologous blood with the potential to release various growth factors (GF) to promote nerve regeneration. This study aims to know the best time for PRP injection to promote nerve regeneration.

**Methods:**

This is an experimental in vivo research using male New Zealand white rabbits in the randomized control group posttest only design. Samples were divided into 5 groups (1 control group and 4 treatment groups). The control group without PRP injection and treated groups injected immediately after nerve injury, 3 days, 7 days, and 14 days afterward. Nerve regeneration was evaluated by the histology specimen sacrificed on day 21. Inflammation cells and endoneurium vacuoles were counted as mean percentage of five nerve fragments in each injured nerve sample specimen.

**Result:**

Inflammation cells and vacuole cells increased significantly when PRP was administered 3 days after injury (group 2) (respectively, 14 ± 6.7 and 56.6 ± 11.6) compared to all treatment groups (*p* < 0.005) (control group, respectively, 6 ± 2.6 and 15.7 ± 9.5). On the other hand, significantly lower endoneurium vacuoles and inflammation cells were found on “the day 14” sample group (respectively, 5 ± 1.3 and 5.2 ± 1.6) compared to all other groups (*p* < 0.005).

**Conclusion:**

This study found that the best time for injecting PRP for nerve regeneration is 14 days after injury.

## 1. Introduction

Peripheral nerve injury is one of the main causes of destruction of the extremities, which cause long-term derangement of physiology. Based on the previous research, the prevalence rate of peripheral nerve injury is approximated at 1.3–2.8%, but if the nerve radix and plexus involved, then the prevalence rate will increase as well. Based on the lesion site, the most commonly reported nerve injury is the upper extremity injury which counts for 61% of the total cases [1]. Morbidity rate caused by peripheral nerve injury is approximately 2.8% of all trauma cases, and the rate depends on the level of nerve injury. Without prompt and proper treatment, prolonged nerve recovery will not only dwindle the possibility of nerve recovery but also cause muscle atrophy and, consequently, permanent loss of muscle function [2–4].

Given the dire consequence of peripheral nerve injuries, appropriate treatment must be employed to help its regeneration. Several types of treatment are available or under development, namely, surgery, growth factor replacement, neuroprotectant (N-acetylcysteine and acetyl-L-carnitine), nerve guidance scaffold for nerve gaps, hormone therapy, and stem cells [5]. Amongst the novel treatment option, platelet-rich plasma (PRP) injection has been found to be a promising method of delivering appropriate growth factors to the injured nerve and consequently help hasten nerve repair [6–8].

PRP is an autologous blood product derived from the processing of the patient's blood which contains high concentration of thrombocytes from normal blood which is suspended in plasma. With a concentration of 1 million thrombocytes/liter in 5 ml plasma, it contains 3–5 times higher growth factors which is useful for nerve regeneration. Previous studies had found PRP does help nerve regeneration in vitro [9–11]. PRP injection is an alternative to mesenchymal stem cells (MSC) as it is easier to produce and is found to provide a comparable effect to nerve regeneration [12].

Despite providing the beneficial effect, its applicability also depends on the timing at which PRP is applied to the injured nerve. PRP has promising results to promote nerve regeneration, but the best time for injection was never studied. A previous study had only studied its effect on nerve regeneration at different nerve evaluation time. Therefore, in this research, we aim to study the difference in the effect of PRP injection on nerve regeneration and to study the best time after the injury to perform the application of PRP.

## 2. Materials and Methods

### 2.1. Trial Conduct

We conducted experimental in vivo research using randomized control group posttest only design. The research was conducted in Animal Hospital Veterinary Faculty of Airlangga University between June and July 2021. Ethical review was granted by Ethical Committee of Veterinary Faculty of Airlangga University (2.KE.040.04.2021 dated 14 April 2021).

### 2.2. Trial Designs and Samples

Included samples were those that fulfill these criteria: male New Zealand white rabbits with axonotmesis sciatic nerve injury, aged 8–12 months, bodyweight of 2400–3200 grams, and healthy (indicated by active movement and normal faeces consistency). Exclusion criteria were samples which died during the experiment (before 21 days), samples who were sick, and those who had an infection on surgical area.

Samples that fulfilled the criteria were first acclimatized for 1 week at the cage in the ITDC laboratory. Samples that exhibit symptoms of sickness were excluded. Afterward, these rabbits were divided into 1 group as control (not-treated) and 4 treatment groups. Each of the treatment group was given PRP injection onto the severed nerve with different delays from nerve injury to PRP application. Name of group and time to treatment is as follows: group 1, immediately after nerve injury; group 2, 3 days after sciatic nerve injury; group 3, 7 days after sciatic nerve injury; group 4, 14 days after sciatic nerve injury. The nerve injury was performed by crushing the nerve with hemostat clamp in an open surgery.

**Figure 1:**
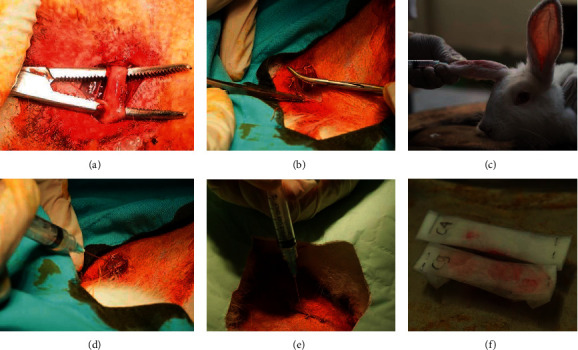
Summary of the sample treatment. (a) Identifying sciatic nerve. (b) Marking and treating nerve lesion using surgical thread. (c) Blood extraction process. (d) Administering PRP immediately after nerve injury (group 1). (e) Administering PRP on day 3 after nerve injury (group 2). (f) Storing process of sample slides after sacrifice; sample slides are wrapped, coded, and stored in formalin.

The samples were anesthetized using intramuscular sulphate atropine 0.2 mg/kg and diazepam 1.0 mg/kg, followed by intramuscular ketamine 20 mg/kg on left quadriceps muscle and maintained with 10 mg/kg ketamine if there was any indication of return to consciousness by the rabbit. The surgery was conducted by first shaving the area of operation and was disinfected using povidone-iodine or chlorhexidine scrub from the sacral to knee area. After preparation, the area was cleaned again using alcohol ethanol 70%. The rabbit was positioned laterally with the back leg abducted and knee in 90° flexion. Using the posterolateral approach, sciatic nerve was identified. The sciatic nerve was then clamped using straight hemostat for 60 seconds to create axonotmesis (crush injury). After compression, injury site was marked using nonabsorbable thread. The marked area would be excised and send for histopathology evaluation after the animal's termination. Afterward, using simple suture with nonabsorbable thread, layer by layer stitch was done to close the surgery incision. Marker for the skin incision was made to indicate the location for PRP subcutaneous injection for the treatment group. Injection of 0.5 ml PRP on the sample was done using 26G needle at the time as planned for each group. For control groups, normal saline was injected instead. The detail of the procedure is shown in Figure 1.

Nerve regeneration was evaluated by the histology specimen sacrificed on day 21. Histopathologic evaluation was conducted on the extracted sample and was stained using hematoxylin and eosin (H&E) stain. Inflammation cells and endoneurium vacuoles were counted as mean percentage from 5 nerve bundle fragments in the injured nerve sample specimen. The inflammation cells and endoneurium vacuoles were counted as cell percentage areas of the microscope's field of view in 200× magnification. Less endoneurium vacuoles and inflammation cells indicate nerve regeneration [13, 14].

### 2.3. Statistical Analysis

We calculated that a sample size needed to provide 90% power with a one-sided error rate of 5% to reject the null hypothesis is 7.

Data were first analyzed descriptively using tables and graphs. Second, data normality and homogeneity were tested using the Shapiro–Wilk test. The data were analyzed using the nonparametric test of Kruskal–Wallis. *P* value of less than 0.05 is accepted as significant.

## 3. Results

### 3.1. Inflammation Cells on Histopathologic Analysis

The amount of detected inflammation cells on the samples are given in Table 1. The highest inflammation cells can be found on group 2 (29 ± 3.3), followed by group 1 (16 ± 11.6), the third one is group 3 (14 ± 6.7), the fourth one is the control group (6 ± 2.6), and least inflammation cells can be found on group 4 (5 ± 1.3).

Statistical analysis is then conducted using the Shapiro–Wilk test. The test resulted as skew distribution of data (*p* < 0.05); therefore, Kruskal–Wallis for the comparation test was used. There was a significant difference between control and other treatment groups. Post hoc analysis was then conducted using the Mann–Whitney test, and the result is given in Table 2. The post hoc analysis showed a significantly higher amount of inflammation cell especially between 3 days after injury group (group 2) with all groups, indicating that this is the worst time for PRP injection to promote nerve regeneration. Compared to the control group, injection on 14 days after injury had the least difference indicating the best time for PRP injection for nerve regeneration.

### 3.2. Endoneurium Vacuoles on Histopathologic Analysis

Amount of detected endoneurium vacuoles are given in Table 3. The highest mean percentage endoneurium vacuoles was found on group 3 (56.6 ± 11.6), followed by group 3 (39.6 ± 25.3), group 1 (33.6 ± 29), and the control group (15.7 ± 9.5). The least amount of endoneurium vacuoles were seen in group 4 (5.2 ± 1.6).

Statistical analysis is then conducted using the Shapiro–Wilk test. The test resulted in skew distribution of data (*p* < 0.05). The comparation test is done using the Kruskal–Wallis test. There was a significant difference between control and other treatment groups. Post hoc analysis was then conducted using the Mann–Whitney test, and the result is given in Table 4.

**Figure 2:**
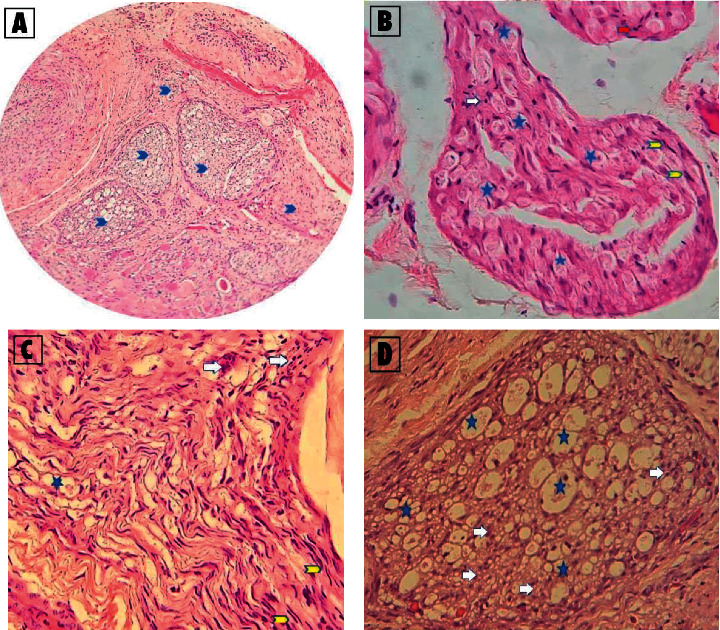
Histopathological evaluation using Hematoxylin Eosin (HE) stain. (A) Five fragments of nerve bundle from 1 sample specimen (100x) from which each bundle would be counted for inflammatory cells and endoneurium vacuole as cell percentage areas (200x). (B) Slides from group 1 with dominant vacuole >30% but less dominant inflammatory cells in 200x magnification. (C) Slides from group 4 with minimum inflammation cells and vacuoles (<5%) in 200x magnification. (D) Slides form group 2 with vacuoles covering almost all of the nerve (>50%) and dominant inflammatory cells (>20%) in 200x magnification. Blue arrow means nerve bundles; blue stars mean vacuoles; yellow arrows mean Schwann cells; and white arrow means inflammatory cells.

The post hoc analysis showed that the amount of endoneurium vacuoles was significantly higher when PRP was given 3 days after injury (group 2) compared to all other groups. This is indicating the worst time for PRP injection to induce nerve regeneration. On the contrary, 14 days after, the injury group (group 5) had significantly lower value compared to all other groups indicating the best timing to inject PRP for nerve regeneration. Even when compared to the control group, injection on 14 days after injury (group 4) has a significant difference. The histopathological evaluation is shown in Figure 2.

## 4. Discussion

Timing at which PRP is injected is based on the sequence of Wallerian degeneration, whereby, on the first 24 hours, nerve degeneration occurs. Until 96 hours or day 3 after injury is when the intense Schwann cell proliferation occurs. Concurrently, until 7 days after injury, removal of degeneration myelin happens, followed by regeneration of the nerve until 14 days after the injury. During these times as well is when the measuring inflammation cells and vacuole cells is a reasonable indicator for nerve regeneration, as it reflects the activity of the recruitment of the macrophages and phagocytosis process [15]. Additionally, these timepoints are chosen because common surgeries are conducted at these timepoints: immediate, 3 days, 7 days, and 14 days [16].

PRP's exact mechanism in peripheral nerve repair is still being extensively studied. One of the most well accepted theory is that PRPs brought growth factors to the injured site. PRP is found to contain various growth factors including platelet-derived growth factor (PDGF), transforming growth factors (TGF-*β*1), platelet-derived angiogenesis factor (PDAF), insulin-like growth factor 1 (IGF-1), platelet factor 4 (PF-4), epidermal growth factor (EGF), epithelial cell growth factor (ECGF), vascular endothelial cell growth factor (VEGF), basic fibroblast growth factor (bFGF), hepatocyte growth factor (HGF), and other cytokines [17]. Over all these factors, FGF plays an important role as it has been found to help axonal outgrowth, neural regeneration, axonal elongation, and neuroprotection through the Nrg1/ErbB, JAK/ STAT 3, or PLCg/Ca2+Oct6 cascade [18].

In this research, we found that PRP is least beneficial in inducing nerve regeneration on group 2 (3 days after injury) and most beneficial in inducing nerve regeneration on group 4 (14 days after injury). Less vacuoles and less inflammatory cells are, in the context of nerve injuries, histopathologic evidence on the acceleration of nerve regeneration [19]. Better outcome after delayed injection of PRP might be since, immediately after injury, there is enough amount of growth factor intrinsically produced. Therefore, addition of growth factors by PRP does not add any beneficial effect. Other possible cause is that PRP helps more in the maturation of nerve instead of initial proliferation as proliferation happens mostly on day 3–7 [16]. Analyzing the PRP's growth factor content before applying it might help in predicting the true net effect of PRP injection as PRP's content itself tend to vary [17].

As to date, within the author's knowledge, there are no similar research which study the timing of PRP injection in relation to nerve regeneration. As such, no direct comparison can be found, but many studies have agreed on the fact that PRP does improve nerve regeneration [19–21]. Despite so, there is a prerequisite for PRPs to work: the injured nerve needs to be in contact or without a gap. With a gap, growth factors effect is found to be significantly less [22, 23]. This might explain why, in this study, PRP needs 14 days after the nerve injury to have a significant effect.

Some factors still need to be considered in regard to timing of PRP injection. First, PRP works through several mechanisms at which it is still not known which mechanism is more dominant at certain days after injury. If this study is the guide, we can conclude that 14 days after injury is the time at which it is most effective, but the exact mechanism is still unclear, and many factors can be the main factors, i.e., growth factor and inflammation suppression. Second, the blood nerve barrier (BNB) might have skewed the effect of PRP. Other studies which study the application of PRP with grafts found that perineurium injection of PRP is better that coating, indicating that the effect of PRP might be dampened by BNB [7, 24]. Other factors might also affect the PRP effect; potential cause of inaccurate result might be due to inaccuracy in the application of PRP itself. Thus, further research needs to use more specific criteria for its sample when determining optimal time for PRP injections, especially regarding length of nerve injury and the site of application.

Other studies studying PRP for nerve regeneration injected PRPs at the same time, but analyzed the nerve regeneration at different times. Moreover, other studies commonly studied the effect of PRP at longer times, commonly after weeks [11, 20, 21]. Research by Abbasipour-Dalivand et al. used behavioral testing, sciatic nerve functional study, and gastrocnemius muscle mass as a nerve recovery parameter and found that PRP does improve nerve regeneration compared to other treatment, but this study does not differ the timing of treatment to injury [21]. Zhu Y et al. studied the effect of different doses of PRP instead and found that PRP with 4.5-fold of blood's concentration resulted in the best nerve regeneration compared to 3.5-fold and 6.5-fold [20]. Research by Ikumi A et al. found that local administration of PRP accelerates SC proliferation and, ultimately, nerve regeneration in vivo [11].

An objective examination that is sufficient to describe the ability of the nerves is electromyography (EMG). EMG determines the distribution of neurogenic abnormalities, focal nerve, or radicular pathology. It can also reveal nerve dysfunction, muscle dysfunction, or problems with nerve-to-muscle signal transmission [25]. In this animal experiment, we could not find the tiny electrodes to translate these signals into graphs, sounds, or numerical values. Thus, this research has several limitations, the absent of electromyographic analysis of the muscle, and the less time used in evaluation interval. Further studies that include electromyographic analysis of the muscle and longer observation period are necessary to ascertain the result of this study.

## 5. Conclusion

This study found that the best time for injecting PRP for nerve regeneration is 14 days after injury. This study can be used as a basis for PRP to be used as an adjuvant therapy for promoting nerve regeneration and for further studies regarding PRP.

## Figures and Tables

**Table 1:** Inflammation cells found on histopathologic analysis on each treatment groups.

	Treatment group (*N*)	Mean ± SD (%)	*P* value
Inflammation cells in mean percentage area of 5 nerve bundle fragments	Control (7)	6 ± 2.6	0.001^a^
	Group 1 (7)	16 ± 11.6	
	Group 2 (7)	29 ± 3.3	
	Group 3 (7)	14 ± 6.7	
	Group 4 (7)	5 ± 1.3	

^a^Kruskal–Wallis.

**Table 2:** Post hoc analysis comparing various groups of treatment with respect to inflammation cells found on histopathologic analysis.

Treatment groups	Mean ± SD	*P* value
Control and immediately after injury	6 ± 2.6 and 16 ± 11	0.149^a^
Control and 3 days after nerve injury	6 ± 2.6 and 29 ± 3.3	0.000^b^^*∗*^
Control and 7 days after nerve injury	6 ± 2.6 and 14 ± 6.7	0.011^b^^*∗*^
Control and 14 days after nerve injury	6 ± 2.6 and 5 ± 1.3	0.589^a^
Immediately after injury and 3 days after nerve injury	16 ± 11 and 29 ± 3.3	0.023^a^^*∗*^
Immediately after injury and 7 days after nerve injury	16 ± 11 and 14 ± 6.7	0.774^a^
Immediately after injury and 14 days after nerve injury	16 ± 11 and 5 ± 1.3	0.084^a^
3 days and 7 days after nerve injury	16 ± 11 and 14 ± 6.7	0.000^b^^*∗*^
3 days and 14 days after nerve injury	16 ± 11 and 5 ± 1.3	0.001^a^^*∗*^
7 days and 14 days after nerve injury	14 ± 6.7 and 5 ± 1.3	0.005^a^^*∗*^

^a^Mann–Whitney. ^b^Independent sample test. ^*∗*^Significant difference (*p* < 0.05).

**Table 3:** Vacuole in cells on histopathologic analysis on each treatment groups.

	Treatment group (*N*)	Mean ± SD (%)	*P* value
Endoneurium vacuoles in mean percentage area of 5 nerve bundle fragments	Control (7)	15.7 ± 9.5 (%)	0.001^a^
	Group 1 (7)	33.6 ± 29 (%)	
	Group 2 (7)	56.6 ± 11.6 (%)	
	Group 3 (7)	39.6 ± 25.3 (%)	
	Group 4 (7)	5.2 ± 1.6 (%)	

^a^Kruskal–Wallis.

**Table 4:** Post hoc analysis comparing various groups of treatment with respect to endoneurium vacuoles found on histopathologic analysis.

Treatment groups	Mean ± SD	*P* value
Control and immediately after injury	15.7 ± 9.5 and 33.6 ± 29	0.149^a^
Control and 3 days after nerve injury	15.7 ± 9.5 and 56.6 ± 11.6	0.002^a^^*∗*^
Control and 7 days after nerve injury	15.7 ± 9.5 and 39.6 ± 25.3	0.003^b^^*∗*^
Control and 14 days after nerve injury	15.7 ± 9.5 and 5.2 ± 1.6	0.023^b^^*∗*^
Immediately after injury and 3 days after nerve injury	33.6 ± 29 and 56.6 ± 11.6	0.023^a^^*∗*^
Immediately after injury and 7 days after nerve injury	33.6 ± 29 and 39.6 ± 25.3	0.774^a^
Immediately after injury and 14 days after nerve injury	33.6 ± 29 and 5.2 ± 1.6	0.020^a^^*∗*^
3 days and 7 days after nerve injury	56.6 ± 11.6 and 14 ± 6.7	0.003^a^^*∗*^
3 days and 14 days after nerve injury	56.6 ± 11.6 and 5.2 ± 1.6	0.002^a^^*∗*^
7 days and 14 days after nerve injury	39.6 ± 25.3 and 5.2 ± 1.6	0.003^b^^*∗*^

^a^Mann–Whitney. ^b^Independent sample test. ^*∗*^Significant difference (*p* < 0.05).

## Data Availability

The data used to support the findings of this study are included within the article.
